# Confirmation of the first report of *Psammolestes tertius* Lent and Jurberg, 1965 (Hemiptera, Reduviidae, Triatominae) in Paraná State, Brazil

**DOI:** 10.1590/0037-8682-0485-2020

**Published:** 2021-02-26

**Authors:** Vinícius Fernandes de Paiva, João Aristeu da Rosa, Walter Ceretti, Mauro Toledo Marrelli, Jader de Oliveira

**Affiliations:** 1 Universidade Estadual de Campinas, Instituto de Biologia, Departamento de Biologia Animal, Campinas, SP, Brasil.; 2 Universidade Estadual Paulista Júlio de Mesquita Filho, Faculdade de Ciências Farmacêuticas, Departamento de Ciências Biológicas, Araraquara, SP, Brasil.; 3 Universidade de São Paulo, Faculdade de Saúde Pública, São Paulo, SP, Brasil.

**Keywords:** Triatomines, Chagas disease, Occurrence

## Abstract

**INTRODUCTION:**

This study confirms the occurrence of *Psammolestes tertius* Lent & Jurberg, 1965 (Hemiptera, Reduviidae, Triatominae) in the state of Paraná, Brazil.

**METHODS:**

In 2002, a male specimen of *P. tertius* was collected in the municipality of Porto Rico, Paraná, Brazil.

**RESULTS:**

This finding adds to the data on the geographical distribution of *P. tertius* from 14 to 15 known occurrences in Brazilian states and, therefore, reports the increase in the diversity of triatomines in Paraná.

**CONCLUSIONS:**

The presence of *P. tertius* in the state of Paraná demonstrates that the biodiversity of these insects may have been underestimated.

The subfamily Triatominae is established as its members are important vectors of *Trypanosoma cruzi* (Chagas, 1909), the etiologic agent of Chagas disease. Currently, the subfamily comprises more than 150 species grouped into 19 genera^1^. The genus *Psammolestes* (Bergroth, 1911) includes three species: *Psammolestes coreodes* (Bergroth, 1911), *Psammolestes arthuri* (Pinto, 1926), and *Psammolestes tertius* (Lent and Jurberg, 1965); however, the role of these species in the epidemiology of human diseases remains uncertain^2^. 

The genus *Psammolestes* has a close association with the nests of some species of birds belonging to the families Dendrocolaptidae, Troglodytidae, Furnariidae, and Icteridae^3^ ^,^ ^4^ ^,^ ^5^ ^,^ ^6^. This close association suggests that birds are their only source of food^2^ ^,^ ^7^.

In the state of Paraná, researchers have reported the occurrence of nine species: *Panstrongylus megistus* (Burmeister, 1835); *Panstrongylus geniculatus* (Latreille, 1811); *Triatoma arthurneivai* (Lent and Martins, 1940); *Triatoma sordida* (Stal, 1859); *Rhodnius neglectus* (Lent, 1954); *Rhodnius domesticus* (Neiva and Pinto, 1923); *Cavernicola pilosa* (Barber, 1937); *Microtriatoma borbai* (Lent and Wygodzinsky, 1979); and, *Triatoma tibiamaculata* (Pinto, 1926)^8^ ^,^ ^9^ ^,^ ^10^. 

The occurence of *P. tertius* has been recorded in Brazil (Minas Gerais, São Paulo, Goiás, Mato Grosso, Pará, Tocantins, Alagoas, Bahia, Ceará, Maranhão, Paraíba, Pernambuco, Piauí, and Rio Grande do Norte) and Peru (San Martín)^10^ ^,^ ^11^ ^,^ ^12^. 

At the Brazilian Congress of Entomology, Silva et al. 2004^13^ presented the first record of *P. tertius* in the state of Paraná. However, according to Article 9.10 of the International Code of Zoological Nomenclature^14^, summary presentations at congresses do not constitute published work; therefore, this summary was not considered by other studies indicating the occurrence of the species in the state of Paraná^6^ ^,^ ^9^ ^,^ ^10^. A male specimen of *P. tertius* (Figure 1) was collected in the municipality of Porto Rico (Figure 2) in a bird’s nest (Furnariidae) on Mutum Island, Paraná River, Paraná, Brazil on February 25, 2002. Voucher specimens can be accessed by request using the registration number CEJMSB 179 corresponding to the entomological collection of Prof. Dr. José Maria Soares Barata, Unesp, Araraquara, São Paulo, Brazil.

**FIGURE 1:**
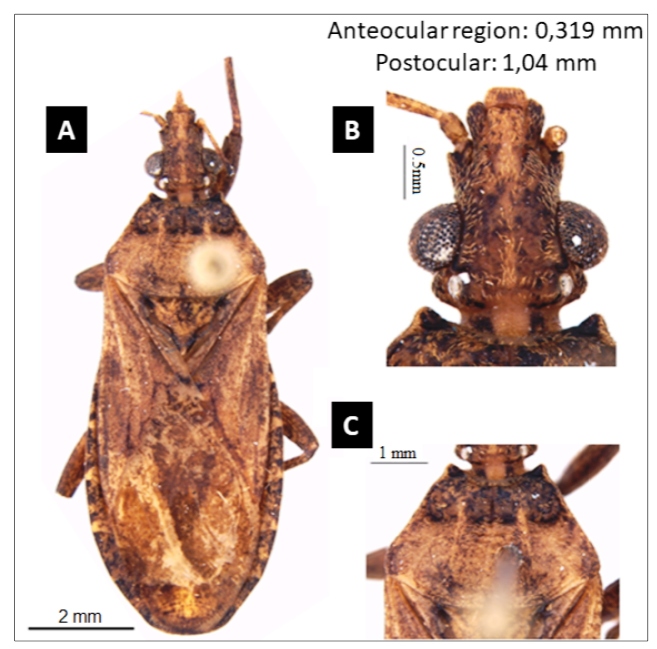
**(A)** Dorsal view of the specimen; **(B)** Detail of the head in dorsal view; and,**(C)** Detail of the pronotum in dorsal view; scale bar: A: 2mm; B: 0.5 mm; and, C: 1mm.

**FIGURE 2:**
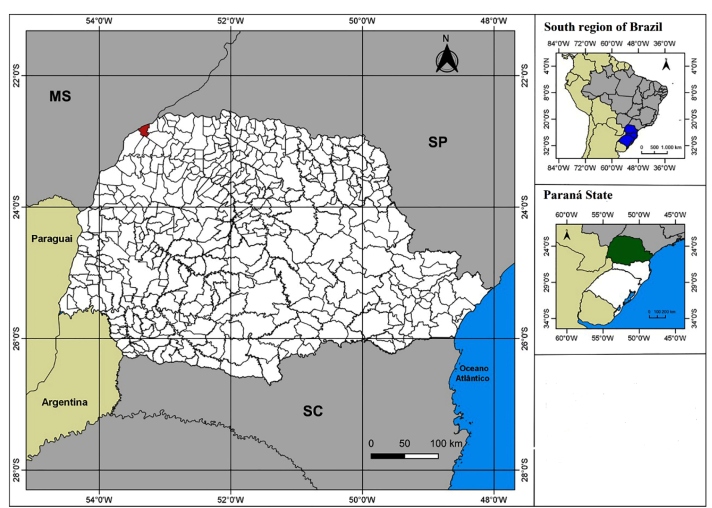
Geographical location of Paraná state (green); Highlighted: Porto Rico, Paraná (Red), Brazil, where *P. tertius* were collected in February 2002. (SC, Santa Catarina state; MS, Mato Grosso do Sul state; SP, São Paulo state).

The identification of the *P. tertius* specimen was conducted in the Laboratory of Parasitology, Faculty of Pharmaceutical Sciences, São Paulo State University (Unesp), Araraquara, São Paulo, Brazil, based on the following criteria: body, small and somewhat dorsoventrally compressed; general color, light yellowish-brown with diffuse darker markings; setae, very short and inconspicuous; head, compressed dorsoventrally, slightly longer than the width at eye level; anteocular region, two or two and a half times the length of the postocular; head on a moderate slope behind the ocelli; anterolateral pronotum angles, very short; hemelytra attaining or almost attaining the apex of the abdomen; legs, short and stout without spines or tubercles (Figure 1)^2^ ^,^ ^6^ ^,^ ^10^.

The genus *Psammolestes* comprises three species and seems to be specialized in exploiting bird-nest ecotopes^2^. Phylogenetic analyses of *P. tertius* and *P. coreodes* suggest that this genus is monophyletic and can be considered a specialized lineage of the *Rhodnius prolixus* group because they share a common ancestor, which in turn highlights the paraphyly of the genus *Rhodnius* ^7^ ^,^ ^15^. Based on a projection of its potential geographic distribution, *P. tertius* presents intermediate climatological limits and occurs at the highest altitudes compared to the other species of the genus^12^. The number of Brazilian states in which *P. tertius* occurred increased from 14 to 15 based on its presence in the state of Paraná, demonstrating that the biodiversity of these insects may have been underestimated and that their role in the epidemiology of Chagas disease remains uncertain.
